# Psychosocial work conditions and traffic safety among minibus and long-bus drivers

**DOI:** 10.1093/joccuh/uiad019

**Published:** 2023-12-28

**Authors:** Mustapha Amoadu, Edward Wilson Ansah, Jacob Owusu Sarfo

**Affiliations:** Department of Health, Physical Education and Recreation, University of Cape Coast, Cape Coast, +233, Ghana; Department of Health, Physical Education and Recreation, University of Cape Coast, Cape Coast, +233, Ghana; Department of Health, Physical Education and Recreation, University of Cape Coast, Cape Coast, +233, Ghana

**Keywords:** psychosocial work factors, traffic safety, road traffic crash, bus drivers

## Abstract

**Objective:** This study sought to examine the association between psychosocial work factors and road traffic crashes (RTCs), and test the differences in psychosocial work factors between minibus and long-bus drivers.

**Methods:** This cross-sectional survey employed a convenient sampling method to collect data from 7315 long-distance minibus and long-bus drivers who operate between the Ghanaian cities, Accra and Tema and other parts of the country. The drivers answered a job content questionnaire, psychosocial safety climate scale (PSC-12), work–family conflict scale, and demographic questions on age, education, driving hours, and RTC history.

**Results:** The correlational analysis showed a significant association between psychosocial work factors and RTCs for the previous 2 years. Hierarchical multiple linear regression found that supervisor support, skill discretion, decision autonomy, psychological demands, PSC, and work–family conflict significantly contributed to explaining RTC rates among the drivers. Also, significant differences were found between minibus and long-bus drivers in driving hours, occurrence of near misses, RTCs, and all psychosocial work factors explored in this study except work–family conflict.

**Conclusions:** Psychosocial work factors directly predict RTCs among minibus and long-bus drivers. Policymakers, driver unions, and owners and managers of bus transport businesses should prioritize integrating occupational health and safety into road transport activities. Furthermore, managers and bus owners should use bottom-up communication, provide access to support services and work-family balance initiatives, flexible work schedules, and a supportive work environment to improve road safety.

## Key points

**What is already known on this topic:** Prior to this study, existing knowledge highlighted the global concern over road traffic crashes (RTCs) and the disproportionately high rates in low- and middle-income countries, including Ghana. There was an understanding of the hazardous working conditions faced by Ghanaian bus drivers, with some evidence linking psychosocial factors to increased RTC risk.

**What this study adds:** This study offers focused insights into the direct association between psychosocial work factors and RTCs among Ghanaian bus drivers, filling a critical void in understanding these relationships within the country’s unique context. It uncovers key predictors such as supervisor support, decision autonomy, and job demands that significantly contribute to explaining RTC occurrences, providing specific, localized knowledge that was previously lacking. Furthermore, the study identifies and differentiates psychosocial work factors between minibus and long-bus drivers, shedding light on disparities within the bus transport sector that impact road safety.

**How this study might affect research, practice, or policy:** The findings highlight the urgent need for integrating occupational health and safety standards into Ghana’s road transport sector, emphasizing the creation of safer and healthier work environments for bus drivers. Insights into psychosocial work factors can inform targeted interventions and policies aimed at reducing RTCs by addressing job demands, providing essential resources, and fostering a better work-life balance.

## 1. Introduction

Road traffic crashes (RTCs) are among the leading causes of death, injury, and disability globally.^1^ The World Health Organization (WHO) has further reported that about 1.3 million people die globally due to RTCs each year, and that low- and middle-income countries (LMICs) account for about 93% of these deaths.^1^ Low-income countries experience a higher rate of 27.5 deaths per 100 000 population compared with high-income countries with 8.2 deaths per 100 000 individuals. Africa bears the highest burden, with a road traffic fatality rate of 26.6 deaths per 100 000 people. The WHO have indicated that these figures are likely underestimates because of the underreporting of such data that is common in developing countries, and challenges in rural data collection.^1^ Unfortunately, the road transport system in Ghana is regarded as one of the most dangerous in Africa.^2^ For instance, evidence indicates that the death rate was 25.7 per 100 000 population in 2019, and about 8 people die every day on Ghana’s roads.^2^^,^^3^ Hence, road safety is of critical public health concern in Ghana.

The WHO has reported that RTCs increase the operational cost of road transport businesses and pose a greater risk to the health and safety of transport operators and other road users.^1^ The road transport sector in Ghana heavily relies on bus services to meet the mobility needs of the population. However, evidence shows that these commercial bus drivers work under precarious conditions, which expose them and other road users to dire health and safety issues.^4^ Studies have found that these precarious working conditions, mostly psychosocial work factors, might explain why bus drivers account for about 36% of road traffic fatalities in Ghana.^4^^,^^5^ Psychosocial work factors are the interactions between job design, work environment, organizational conditions, and employees’ capabilities.^6^ In the road transport sector, such factors may include long driving hours, job autonomy, skill discretion, job insecurity, work–family conflict, high physical and psychological work demands, lone driving, and irregular shift work.^4^

Exposure of drivers to precarious work without essential job resources for coping may lead to impaired health and safety outcomes, including RTCs.^6^ This argument is highlighted by key work stress theories and models such as the effort-reward imbalance model, job-demand-control model, job-demand-resource model, and the psychosocial safety climate (PSC) theory.^7^^–^^10^ These work stress models hold that a stressful work environment is created when high job demands are not matched with relevant job resources like skill discretion, autonomy, and support from supervisors and co-workers.^7^ The stressful work environment of commercial drivers may not only affect drivers’ well-being and safety, but also defeats the purpose of safe work values that seek to eliminate all forms of precarious work.^11^^,^^12^ Beyond that, it creates public health concern where many other road users die, get injured, or become disabled because of road accidents.^4^^,^^5^ The economic cost to the individuals, their families, transport companies, and the nation is huge.

Furthermore, a recent scoping review^13^ found that psychosocial work factors such as job demands and job resources are predictors of RTCs and risky driving behaviors. For instance, high job demands like emotional, psychological, and physical demands are associated with increased occurrence of RTCs.^6^^,^^14^^,^^15^ On the other hand, a lack of social support from co-workers and supervisors is a risk factor for RTCs.^6^^,^^15^^–^^18^ Other psychosocial work factors, such as work–family conflicts^18^ and job insecurity, have been reported as significant predictors of risky driving behaviors leading to RTCs.^6^

Despite the importance of traffic safety to drivers, there is a significant research gap in the road transport sector in Ghana, particularly concerning the role of psychosocial work factors in explaining the occurrence of RTCs. Thus, although there is some evidence from the international community linking psychosocial work factors to traffic safety outcomes among bus drivers,^6^^,^^15^^,^^17^^,^^18^ little is known about the specific context of commercial drivers in Ghana. This lack of evidence explains why most road safety initiatives in Ghana have ignored the importance of drivers’ working conditions.^4^ Hence, it is important to investigate the relationship between psychosocial work factors and traffic safety among bus drivers in Ghana to identify the specific factors within the work environment of commercial bus drivers that may account for the rise in RTCs.

Studies examining psychosocial work factors often include a variety of driver groups, ranging from taxi drivers, bus operators, and rapid bus transit drivers to hauliers, tricycle operators, bus conductors, and drivers of hazardous vehicles.^13^ This circumstance creates a challenge when establishing policies to effectively capture the operational and contextual uniqueness of the activities of these driver groups. Even within the same driver group, such as bus drivers, disparities in working conditions may exist. For example, although minibus and long-bus drivers have comparable work schedules, like operating over long distances, their psychosocial work factors might differ regarding their activities and work environment.^6^ Studies have also indicated that minibus drivers in Ghana are notorious for reckless driving and have a high incidence of on-the-road-crashes.^5^^,^^19^

Nevertheless, the working conditions of drivers in developing countries like Ghana have not been thoroughly investigated. Therefore, comparing the psychosocial work factors of minibus and long-bus drivers is critical for policy and other safety interventions. Addressing the research gap we have identified may help provide a comprehensive understanding of the impact of psychosocial work factors on traffic safety among minibus and long-bus drivers. The results of the study may have practical implications for improving road safety and creating safe, decent, and healthy work environments for bus drivers and reduce the carnage on Ghana’s roads. Therefore, this study aimed to examine the association between psychosocial work factors and RTCs, and test the differences in psychosocial work factors for minibus and long-bus drivers in Ghana.

## 2. Methods

### 2.1. Design and participants

This cross-sectional survey studied 7315 commercial bus drivers who work for individual and public bus companies in Accra and Tema (Ghana). Using a convenient sampling method, the participants comprised 5260 (71.9%) minibus drivers and 2055 (28.1%) long-bus drivers. Thus, we recruited minibus drivers and long-bus drivers who were readily available at their stations or bus terminals and were willing to participate in the study. These long-distance bus drivers commute at least 140 km or 3 hours^19^ on a single trip from Accra and Tema to other parts of the country and/or to other major cities in West Africa.

### 2.2. Measures

A 27-item job content questionnaire (JCQ)^20^ was used to measure supervisor support (4 items, α = .913), co-worker support (4 items, α = .857), skill discretion (6 items, α = .926), decision authority (4 items, α = .725), psychological demands (6 items, α = .950), and job insecurity (3 items, α = .830). Some of these items included, *“My job requires hard work all the time”* and *“People I work with are competent to do their job.”* The JCQ is scored using a 4-point Likert scale, from strongly disagree (1) to strongly agree (4), where higher scores indicate higher job demands and job resources.

Work–family conflict was measured using the work–family conflict scale,^21^ a 5-item scale deemed reliable (α = .764). “*My work has a negative impact on my family life”* is an item on the scale. Responses range from strongly disagree (1) to strongly agree (4), where a high score denotes a higher risk of conflict.

PSC was measured using PSC-12^22^ with a reliability value of .94. *“In my workplace, senior management/car owner acts quickly to correct problems/issues that affect employees’ psychological health,”* is an item on the PSC-12, with response options of strongly disagree (1) to strongly agree (4). A high PSC score indicates that bus drivers are involved in decision making at their workplaces, and their well-being and safety are prioritized by their management, supervisors, or car owners.

The bus drivers also responded to brief sociodemographic items like age, educational background, daily working hours, and weekly working days. Also, near misses and RTCs recorded for 2 years before data collection were taken from the drivers.

### 2.3. Procedures and ethics

The bus drivers were invited to this survey through their “station masters” (a Ghanaian term for transport station managers) and bus terminal administrators. Drivers who had returned from their trips or were waiting (at their terminals) for the next trip were sampled for this study. Survey interviews were conducted for the bus drivers who voluntarily accepted and consented to participate in the study. The questionnaire was translated into Twi (the most popular local dialect in the study area) and translated back into English, to ensure translation accuracy. The survey interviews lasted 15 to 30 minutes. Twenty trained field assistants administered the survey interviews, which were conducted over 2 months (November 28, 2023 to January 30, 2023).

Bus drivers who participated in the study were informed about their rights to withdraw from the study at any time. We assured the drivers of the protection of their information. Furthermore, they signed or gave oral informed consent before taking part in the study. Bus drivers who took part in the study were informed that the survey was solely for research purposes. Participants were not compensated in cash or kind. We also obtained ethical approval from the University of Cape Coast Institutional Review Board (ID: UCCIRB/CES/2022/82) before data collection commenced. There was a 95.9% response rate.

**Table 1:** Means, SDs, and the range of the study variables.

	**Study variables**	**Range**	**Mean**	**95% CI (lower-upper)**	**SD**	**95% CI (lower-upper)**
**1**	**Age, y**	19-64	39.11	38.93-39.28	7.91	7.79-8.03
**2**	**RTCs (last 2 y)**	0-5	1.02	0.99-1.05	1.17	1.15-1.19
**3**	**Near misses (last 2 y)**	0-23	3.80	3.73-3.87	3.20	3.08-3.31
**4**	**Weekly driving days**	4-7	5.68	5.66-5.70	0.86	0.85-0.88
**5**	**Weekly working hours**	20-126	53.77	53.28-54.28	22.47	22.07-22.85
**6**	**Supervisor support**	12-16	12.40	12.37-12.42	1.06	1.03-1.10
**7**	**Co-worker support**	9-16	12.42	12.40-12.44	1.03	1.00-1.05
**8**	**Skill discretion**	6-24	13.32	13.20-13.44	5.04	4.98-5.11
**9**	**Decision autonomy**	3-9	5.72	5.67-5.76	1.76	1.74-1.78
**10**	**Psychological demands**	11-20	14.95	14.89-15.02	2.87	2.85-2.89
**11**	**Job insecurity**	5-12	10.00	9.96-10.03	1.51	1.48-1.53
**12**	**Work–family conflict**	4-21	16.81	16.72-16.88	3.55	3.49-3.61
**13**	**PSC**	23-46	35.57	35.47-35.67	4.17	4.07-4.27

PSC, psychosocial safety climate; RTC, road traffic crash.

### 2.4. Statistical analysis

Descriptive and correlation analyses were conducted to establish the potential relationship between psychosocial work factors and RTCs. Furthermore, hierarchical multiple regression was used to predict bus drivers’ RTCs recorded in the 2 years previous to data collection. Sociodemographic variables (educational background and age) and weekly driving intensity were first entered into the hierarchical multiple regression (Model 1). Daily working hours were multiplied by the weekly working days to calculate weekly driving intensity. Psychosocial work factors (supervisor support, co-worker support, decision autonomy, skill discretion, psychological demands, job insecurity, work–family conflict, and PSC) were then entered into the second model (Model 2). In addition, an independent *t*-test was conducted to compare minibus and long-bus drivers on psychosocial work factors, driving intensity, and RTCs. Compliance with the minimum parameters of each statistical test was analyzed. Data management was conducted using IBM Statistical Package for Social Sciences version 27.

## 3. Results

### 3.1. Descriptive data

The age range for these bus divers was 19-64 years, with a mean (SD) age of 39 (7.85) years. The years of driving experience ranged between 1 and 47 years. Most (49.9%) of these bus drivers had basic education, 13% had no formal education, and only 3.1% had tertiary education. Unfortunately, the drivers reported an average of 1.07 RTC (SD = 0.1.25; range 0-5) in the 2 years previous to data collection.

### 3.2. Correlational analysis

Tables 1 and2 present the descriptive and correlational analysis, respectively. The age of the bus drivers had a positive and significant association with supervisor support, co-worker support, decision autonomy, skill discretion, and PSC. Furthermore, age had a significant negative association with psychological demands, job insecurity, work–family conflict, weekly hour intensity, RTCs, and near misses. Psychological demands, job insecurity, work–family conflict, and weekly driving hours had a significant positive association with RTCs and near misses. Moreover, skill discretion, decision autonomy, supervisor support, co-worker support, and PSC had significant negative associations with RTCs as reported by the drivers. Decision autonomy had a significant positive association with near misses.

**Table 2:** Correlation (Pearson *r*) between study variables.

	**Study variables**	**1**	**2**	**3**	**4**	**5**	**6**	**7**	**8**	**9**	**10**	**11**	**12**	**13**
**1**	**Age, y**	—												
**2**	**RTCs (last 2 y)**	−0.750^**^	—											
**3**	**Near misses (last 2 y)**	−0.768^**^	0.613^**^	—										
**4**	**Weekly driving days**	0.023	−0.006	0.001	—									
**5**	**Weekly working hours**	−0.181^**^	0.128^**^	0.148^**^	0.363^**^	—								
**6**	**Supervisor support**	0.045^**^	−0.095^**^	−0.140^**^	−0.016	0.018	—							
**7**	**Co-worker support**	0.087^**^	−0.127^**^	−0.163^**^	−0.018	−0.002	0.880^**^	—						
**8**	**Skill discretion**	0.691^**^	−0.703^**^	−0.583^**^	−0.001	−0.194^**^	0.002	0.058^**^	—					
**9**	**Decision autonomy**	0.166^**^	−0.202^**^	0.089^**^	−0.013	−0.188^**^	−0.355^**^	−0.319^**^	0.220^**^	—				
**10**	**Psychological demands**	−0.540^**^	0.584^**^	0.451^**^	0.014	0.153^**^	0.146^**^	0.047^**^	−0.661^**^	−0.118^**^	—			
**11**	**Job insecurity**	−0.594^**^	0.577^**^	0.547^**^	−0.012	0.136^**^	−0.107^**^	−0.157^**^	−0.820^**^	−0.113^**^	0.631^**^	—		
**12**	**Work–family conflict**	−0.054^**^	0.025^**^	0.030^*^	−0.012	0.005	0.045^**^	0.004	−0.013	0.001	−0.051^**^	0.044^**^	—	
**13**	**PSC**	0.042^**^	−0.031^**^	0.152^**^	0.017	−0.040	−0.840^**^	−0.768^**^	0.017	0.486^**^	−0.043^**^	0.159^**^	−0.051	—

^*^Correlation is significant at the .05 level (2-tailed).

^**^Correlation is significant at the .01 level (2-tailed). PSC, psychosocial safety climate; RTC, road traffic crash.

### 3.3. Multiple regression analysis

Table 3 presents the results of the hierarchical multiple linear regression analysis. The results indicated the existence of an association between psychosocial work factors and RTCs, as reported by the drivers. In Model 1, age, education level, and weekly driving intensity explained 50.6% (adjusted *R*^2^ = 0.506) of variance in RTCs. The addition of the psychosocial work factors to the model (Model 2) explained an additional 12% of variance (change in adjusted *R*^2^ = 0.120) in RTCs. Thus, in Model 2, the variables explained 62.5% of the variance in RTCs, which indicates a high predictive power of the regression model. Besides, there was a low mean square error of 0.033, which indicates that the model’s prediction has a relatively low error.

**Table 3:** Hierarchical multiple linear regression coefficients of predictors of RTCs.

**Model**	**Variables**	**Adjusted *R*** ^**2**^	**Change in adjusted *R*** ^**2**^	**Unstandardized coefficients**		**Standardized coefficients**	**Significance**	**Tolerance**	**VIF**
				**B**	**Standard error**	**Beta**			
**1**	**(Constant)**	0.506	0.506	5.180	0.066				
	**Age**			−0.102	0.002	−0.638	.000	.752	1.329
	**Educational level**			−0.126	0.011	−0.101	.000	.797	1.254
	**Weekly driving hours**			0.098	0.012	0.069	.000	.927	1.079
									
**2**	**(Constant)**	0.625	0.120	10.751	0.354				
	**Age**			−0.056	0.002	−0.351	.000	.442	2.261
	**Educational level**			−0.065	0.010	−0.052	.000	.772	1.295
	**Weekly hour intensity**			0.010	0.011	0.007	.370	.876	1.141
	**Supervisor support**			−0.472	0.023	−0.397	.000	.140	7.165
	**Co-worker support**			0.016	0.019	0.013	.404	.214	4.679
	**Psychological demands**			0.119	0.005	0.270	.000	.461	2.167
	**Skill discretion**			−0.042	0.004	−0.169	.000	.241	4.145
	**Decision autonomy**			−0.080	0.006	−0.113	.000	.689	1.451
	**Job insecurity**			0.020	0.011	0.024	.074	.281	3.563
	**Work–family conflict**			0.015	0.003	0.041	.000	.965	1.037
	**PSC**			−0.081	0.005	−0.267	.000	.213	4.695

B, unstandardized coefficient beta; PSC, psychosocial safety climate; RTC, road traffic crash; VIF, variance inflation factor.

Dependent variable: road traffic crash (last 2 years).

The assumptions of regression were examined using the recommendations of Osborne and Waters^23^ and Williams et al.^24^ Partial plots and scatter plots of the predictors in the model confirmed that the assumption of linearity and homoscedasticity were not violated. Also, high tolerance values >0.1 and variance inflation factors values <10 were found, which further indicated that issues of multicollinearity did not exist among the variables in the regression model.^23^ Also, with a Durbin-Watson statistic of 1.954, it is assumed that issues of autocorrelation did not exist in the data.

We detected a violation of the normality of the residual distribution assumption (Kolmogorov-Smirnov = 0.267, *P* = .000; Shapiro-Wilk = 0.645, *P* = .000), which suggestes that caution is needed when interpreting the results. However, according to William et al,^24^ when other assumptions of regression are met, compliance with the assumption of normally distributed errors is not needed to obtain a reliable and unbiased coefficient of regression. Besides, in a sample greater than 200, the central theorem guarantees that residual distribution approaches a normal distribution.^23^^,^^24^ Hence, regression models are relatively robust to the normal residual distribution assumption.^24^

### 3.4. Comparisons between the minibus and long-bus drivers

Table 4 presents the analysis that compares minibus and long-bus drivers on psychosocial work factors, driving hour intensity, and safety incidents (RTCs and near misses). The results show that co-worker and supervisor supports were significantly higher among minibus drivers than long-bus drivers. Decision autonomy, skill discretion, and PSC were also significantly higher among long-bus drivers. Moreover, psychological demands, job insecurities, and work–family conflict were significantly higher among minibus drivers. Thus, minibus drivers had higher levels of job demands compared with long-bus drivers. Furthermore, minibus drivers reported significantly higher daily and weekly driving hour intensity. Finally, minibus drivers reported higher levels of near misses and RTCs compared with long-bus drivers within the previous 2 years.

**Table 4:** Descriptive analysis of psychosocial work factors, RTCs, and near misses by type of bus.

**Factor**	**Minibus**		**Long bus**		***t* statistic**	**Significance**
	**Mean**	**SD**	**Mean**	**SD**		
**Co-worker support**	12.4473	1.06004	12.3564	.93612	3.574	.000
**Supervisor support**	12.4518	1.12968	12.2711	.86596	7.289	.000
**Decision autonomy**	5.3779	1.68344	6.5597	1.65889	-27.155	.000
**Skill discretion**	11.5541	4.40086	17.7318	3.64180	-61.046	.000
**Psychological demands**	15.7184	2.89040	13.0346	1.66610	49.149	.000
**Work–family conflict**	16.8433	3.43993	16.7148	3.81641	1.325	.185
**Job insecurity**	10.4531	1.23477	8.8537	1.52285	42.327	.000
**PSC**	35.1740	3.90345	36.5480	4.61440	-11.891	.001
**Daily driving hours**	9.8414	3.55220	8.5173	3.61434	14.197	.001
**Weekly driving hours**	55.7548	21.88843	48.7889	23.14227	11.982	.001
**Accidents**	1.4200	1.15823	.0180	.17988	84.201	.000
**Near misses**	4.6843	3.36495	1.5900	.83115	61.354	.000

PSC, psychosocial safety climate; RTC, road traffic crash.

## 4. Discussion

The objective of this study was to investigate the correlation between psychosocial work factors and RTCs among bus drivers, and analyze variations in psychosocial work factors, driving hours, and safety incidents (near misses and RTCs). This study was conducted to direct the attention of policymakers and researchers to the working conditions of bus drivers in Ghana, which have been ignored. This study was also conducted to generate knowledge focused on risk and protective factors that transcend the individual drivers to a social work environment, job design, and organizational conditions, which have been the contemporary focus of occupational health and safety literature.^13^^,^^16^^,^^25^ The results on the association between psychosocial work factors and RTCs support the assumptions of work stress models.^7^^,^^26^ In the regression model it was found that several psychosocial work factors such as supervisor support, decision autonomy, skill discretion, psychological demands, work–family conflicts, and PSC predicted the occurrence of RTCs, as also reported in previous studies.^13^^,^^16^^–^^18^^,^^25^ For instance, job demands, job resources, and work–family conflicts are associated with RTCs among rapid bus transit operators, intercity bus drivers, and other professional drivers in Colombia^6^^,^^15^^,^^17^^,^^25^ and Malaysia.^18^

### 4.1. Job resources and RTCs

The negative effects of PSC, supervisor support, decision autonomy, and skill discretion on RTCs indicated that providing commercial bus drivers with supportive supervision, feedback, adequate training in safety, job control, reasonable schedules, and bottom-up communication may help reduce RTCs among drivers in Ghana. For instance, within a high PSC environment, bus drivers may be motivated to report safety incidents and challenging work schedules via bottom-up communication channels. Also, in a high-resource work context, these drivers are motivated to participate in job design and safety practices, known for reducing workplace accidents and injuries^7^^,^^25^ that create public health challenges via injuries, disabilities, and death, even among other road users.

In practice, a driver with high decision autonomy, in a high PSC work context, has the confidence to utilize available job resources to lessen the impact of high job demands on their driving^7^^,^^16^ For example, with high supervisor support, bus drivers are more likely to succeed in seeking the support of management or individual bus owners in managing their workload and driving hour intensity.^27^^,^^28^ Then, moderating drivers’ workload in the Ghanaian context may include reducing daily or weekly trips and sales targets or recruiting supporting staff like conductors and co-drivers. Furthermore, in a high job resource context, drivers would have access to recovery periods after long trips and take regular rest breaks during long driving shifts, for improved driving performance and driver well-being.^16^^,^^29^ Notably, a high job resource context indicates that the well-being and safety of the drivers are prioritized.^13^^,^^30^^,^^31^ This is one of the ways of eliminating precarious work conditions and reducing RTCs in the Ghanaian road transport sector. A high job resource context may further indicate that practical efforts are being made to create safe and decent work for this vulnerable occupational group.

Co-worker support did not make a statistically significant contribution to explaining RTCs among bus drivers in Ghana. This result contradicts the findings of a previous study conducted among bus drivers in Colombia, where co-worker support had a significant impact on road crashes.^6^ This indicates that co-worker support may have a limited direct effect on driving behaviors among these drivers in Ghana. The role and importance of co-worker support in road transport activities may vary across different cultural contexts. In the Ghanaian bus transport sector, co-workers are mostly bus conductors who may be learning how to drive and, hence, make a limited contribution to reducing on-the-road risky behaviors and driving hour intensity of the drivers. Also, most long-bus drivers in Ghana drive alone, without the support of bus conductors, and this may affect their perception of the importance of co-workers.^32^ Hence, the cultural norms, work dynamics, and social hierarchies within the bus transport industry in Ghana affect how co-worker support can control the occurrence of RTCs.

### 4.2. Job demands and RTCs

Psychological demands and work–family conflicts have significant positive contributions to explaining RTCs among the drivers. Previous studies have reported this finding among bus and taxi drivers in Colombia^6^ and Malysia.^14^^,^^18^ Psychological demands and work–family conflict have led to increased levels of stress among the bus drivers.^18^ These high stress levels negatively affect the concentration, attention, and decision-making abilities of the drivers, increasing the likelihood of on-the-road errors and violations^14^ with attendant implications. For example, worrying about family issues may divert a driver’s attention from the road, further increasing risky driving behaviors leading to an RTC.^14^ Besides, a compromised work-life balance could result in fatigue, sleep disturbances, and reduced psychological resilience that would impair driving performance. Therefore, implementing policies such as providing flexible work schedules, promoting work-family life balance, adopting bottom-up communication, and promoting stress management programs would help deal with the increasing job demands and work–family conflict among this group of drivers. We found that job insecurity did not make a significant contribution to RTCs, contrary to a finding in a previous study.^14^ Perhaps the bus drivers are not too worried about job losses. This warrants further research study.

### 4.3. Differences in psychosocial work factors by bus driver groups

Minibus drivers had significant levels of supervisor and co-worker support compared with long-bus drivers. In the Ghanaian bus transport industry, minibuses are predominantly owned by individuals who are actively involved in running the business to increase productivity, safety of the drivers, and the vehicle. Thus, minibus drivers are more likely to recruit a bus conductor because they usually make frequent stops during trips to offload or onboard new passengers. In the Ghanaian context, minibus drivers are likely to seek the support of co-drivers, known locally as “spare drivers,” to enable the drivers to have adequate recovery periods and rest breaks. These spare drivers do not exist in the work environment of long-bus drivers, where buses are predominantly owned by private companies or the state, making the effect of an organizational variable, like social support, less paramount.

We found that PSC was significantly higher among long-bus drivers compared with minibus drivers. PSC is an upstream organizational or job resource that is more likely to be perceived by drivers in well-structured or formal organizations, as in the long-bus transport businesses in Ghana.^7^ This may mean that minibus drivers report a significantly greater perception of psychological demands and job insecurity. The work activities of minibus drivers are predominantly informal where job insecurity is high due to high levels of daily sales or targets by bus owners. This situation may force minibus drivers to drive long hours to meet precarious employment contract demands. Therefore, minibus drivers may experience and report high levels of RTCs because such drivers work under more precarious conditions. Unfortunately, minibus driving conditions are also informal in nature and nonstructured, with lack of controls and low integration of occupational health and safety standards.

### 4.4. Practical implications for policy

In developed industrialized nations, workplace fatalities and injuries, including road traffic accidents, have seen reductions due to stringent regulations, effective policies, robust interventions, and the incorporation of occupational health and safety into the road transport operations.^25^ However, in developing countries, like Ghana, the realm of occupational health and safety remains relatively nascent,^33^ leading to high rates of road transport accidents and associated casualties that are challenging to address. Despite the existence of regulations that govern driving hours and rest periods in Ghana, the practical enforcement of these road traffic regulations and safety standards for guiding road transport activities and operators faces significant challenges.^1^^,^^34^^–^^36^ To enhance driver well-being, and reduce carnage on the road and attendant socioeconomic and human costs, it is imperative for bus transport companies, driver unions, and the Ghana Road Safety Commission to integrate occupational health and safety standards in the road transport sector. Strategic investments and concerted efforts are essential for the successful implementation of road traffic regulations. Drivers’ working conditions need to be given the necessary policy, research, and provision of resources to help create a safe and decent work environment where precarious working conditions are eliminated. Bus transport owners and businesses should involve drivers in their job design and adopt the bottom-up communication approach that encourages drivers to give feedback and ensure work stressors are thoroughly discussed and managed.

### 4.5. Study limitations and recommendations for future studies

This was a cross-sectional survey, so the findings and conclusions do not imply cause-and-effect relations. Also, the use of self-response measures may have introduced some level of response bias. Moreover, collecting accident data from up to 2 years previously may have introduced recall bias that is likely to underestimate the rate of RTCs. Furthermore, the violation of the assumption of normal distribution of errors means that interpretation and generalization of findings should be done with cautions. However, this study used a large sample, standardized measures, and robust statistical tools that help make robust conclusions and recommendations based on the findings. Perhaps the use of complementary objective measures, such as secondary data on the accident history of bus drivers, may be useful. More studies are needed to understand the working conditions of other driver groups, and the use of qualitative designs to gain in-depth understanding of how precarious working conditions affect drivers’ performance, well-being, and RTCs is warranted.

### 4.6. Conclusions

The data in this study suggest a significant relationship between psychosocial work factors and RTCs from the previous 2 years. Furthermore, our data suggest that psychosocial work factors such as PSC, supervisor support, decision autonomy, skill discretion, psychological demands, and work–family conflict contributed to explaining the occurrence of RTCs among long-distance bus drivers in Ghana. Moreover, statistically significant differences were found between minibus and long-bus drivers on driving hours, near misses, RTCs, and all psychosocial work factors except work–family conflict. Therefore, policy makers, driver unions, and bus transport owners and companies should prioritize integrating occupational health and safety into road transport activities. Furthermore, using bottom-up communication, providing access to support services and work-family balance initiatives, flexible work schedules, and a supportive work environment would help improve road safety among long-distance bus drivers in Ghana. This will be a good step towards creating a safe, decent, and healthy workplace for all and to limiting on-the-road accidents and their associated high costs to the nation.

## Author contributions

All authors were responsible for conceptualization and methodology; data collection, analysis, and writing of the original draft; and review and editing.

## Funding

This research did not receive any specific grant from funding agencies in the public, commercial, or not-for-profit sectors.

## Conflicts of interest

We declare no conflicts of interest for this article.

## Data availability

The data that support the findings of this study are available from the corresponding author upon reasonable request.
